# The clinical efficacy of Shi-style lumbar manipulations for symptomatic degenerative lumbar spondylolisthesis: protocol for a randomized, blinded, controlled trial

**DOI:** 10.1186/s13018-019-1214-x

**Published:** 2019-06-14

**Authors:** Mengchen Yin, Jie Ye, Ruirui Xue, Liang Qiao, Junming Ma, Wen Mo

**Affiliations:** Department of Orthopaedics, LongHua Hospital, Shanghai University of Traditional Chinese Medicine, Shanghai, China

**Keywords:** Chiropractic manipulations, Degenerative lumbar spondylolisthesis, Shi-style lumbar manipulation, Mechanical lumbar traction, Randomized controlled trial

## Abstract

**Background:**

Symptomatic degenerative lumbar spondylolisthesis (DLS) presents spinal problems in daily life. Shi-style lumbar manipulation (SLM), as an alternative treatment for DLS, is popular in China. SLM is based on the channels and collaterals theory of the traditional Chinese medicine, in which the symptoms are believed to result from channel blockage and joint displacement. However, there is no solid evidence to show the effect of the SLM on the management of symptomatic DLS.

**Methods/design:**

We conduct a prospective randomized, blinded, controlled trial to compare the effectiveness of SLM with mechanical lumbar traction and explore whether it could be a potential therapy for symptomatic DLS. A total of 60 patients with symptomatic DLS will be enrolled and treated with the SLM or mechanical lumbar traction for 2 weeks. VAS score and SF-36 questionnaire were assessed at baseline and at 2, 4, 12, and 24 weeks. Any signs of acute adverse reactions, such as lower limb paralysis or syndrome of cauda equina, will be recorded at each visit during treatment.

**Discussion:**

Although the SLM has been used in China for many years to treat symptomatic DLS, there is a lack of consensus about its effectiveness. This trial will provide convincing evidence about the effect of SLM on symptomatic DLS.

**Trial registration:**

Registered on 6 January 2019; the trial number is ChiCTR1900020519.

**Electronic supplementary material:**

The online version of this article (10.1186/s13018-019-1214-x) contains supplementary material, which is available to authorized users.

## Introduction

Symptomatic degenerative lumbar spondylolisthesis (DLS) presents spinal problems in daily life [1]. The osteoarthritic and degenerative changes in disc, facet joints, and ligament cause spinal stenosis and result in mechanical low back pain and radiating pain in buttock and legs. Currently, degenerative lumbar spondylolisthesis is treated with both surgical and non-surgical methods. Surgical intervention is applied for patients with severe pain or progressive neurologic deficits. The non-surgical/conservative management (e.g., NSAIDS drugs, physical therapy, or chiropractic manipulations) is usually applied in the first instance [2, 3].

Shi-style lumbar manipulation (SLM), as an alternative treatment for DLS, is popular in China. SLM is based on the channels and collaterals theory of the traditional Chinese medicine, in which the symptoms are believed to result from channel blockage and joint displacement [4, 5]. Despite its popularity in China, few studies have investigated the effectiveness of Shi-style manipulations in the management of chronic low back pain. Therefore, this prospective, randomized, controlled clinical trial aims to compare the effectiveness of SLM with mechanical lumbar traction and explore whether it could be a potential therapy for symptomatic DLS.

## Materials and methods

### Study design

**Fig. 1:**
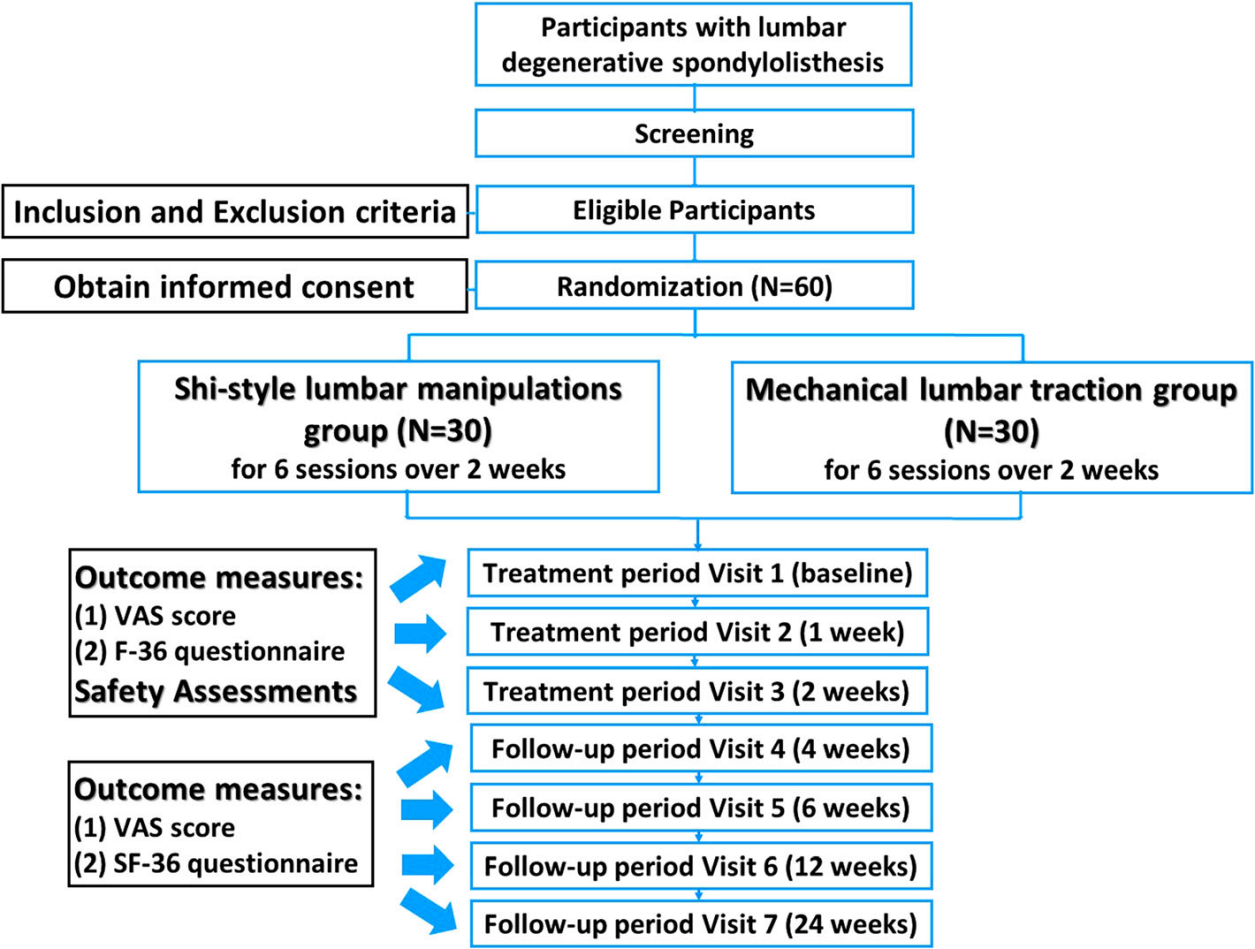
Study flow

This study is a prospective, outcome assessor-, and data analyst-blinded randomized controlled clinical trial. The objective of this proposed study is to investigate whether SLM could lead to an improvement in DLS patients. The principal investigator (PI) is responsible for the overall project and organizing of steering committee meetings. PIs of sub-center departments are responsible for gathering experts to carry out the project. An independent steering committee will be responsible for affairs such as participants’ safety, meetings, recruitment and follow-up of participants, and quality control. The coordinating center is responsible for communicating protocol modifications and providing materials. This trial includes a 2-week treatment period and a 6-month follow-up period. After randomization, patients will receive 6-session treatment over a period of 2 weeks. Outcome assessments will be conducted at baseline, as well as at 2, 4, 12, and 24 weeks (Fig. 1; Additional file 1).

### Eligibility criteria

#### Inclusion criteria

Participants who meet the criteria below are eligible [6, 7].

- Adults (aged ≥ 18 years) with DLS
- Meeting the diagnostic criteria for DLS (The North American Spine Society, NASS)
- Having persistent symptoms for more than 12 months before enrollment
- Being willing to give informed consent

#### Exclusion criteria

The exclusion criteria are as follows [6, 7]:

- DLS combined with neurological diseases, such as polio, Guillain-Barre syndrome, and myasthenia
- Imaging examination indicating pedicle fissure, tuberculosis, or spine fracture
- DLS combined with systemic diseases, such as malignant tumors, diabetes, severe rheumatism, or severe osteoporosis
- Lumbar surgery before enrollment
- Failure to understand or sign informed consent

### Patient population and recruitment procedure

Participants will be recruited from Longhua Hospital affiliated with the Shanghai University of Traditional Chinese Medicine. Prospective participants will be interviewed by the coordinators and informed of the eligibility criteria and the procedure. Those who are eligible and willing to participate in the study will be screened initially by baseline assessment and then diagnosed based upon clinical manifestations, physical examination, and imaging. Participants will be informed that participating in the trial was absolutely voluntary and withdrawal from the trial can be made at any time. In case of withdrawal, the data collected will not be deleted and will be used in the final analyses. A data compilation form including all variables of interest and all potential risks will be completed by the corresponding research center. The information obtained will be stored in an electronic database for subsequent statistical analysis. Recruitment will start in January 2019 and is expected to end in July 2020. The final follow-up of all participants will be completed on 31 December 2020. The overview of the participant processing and the schedule of evaluation is provided in Fig. 1.

### Ethics

This study will be conducted in accordance with the principles of the Declaration of Helsinki for Clinical Research [8]. The trial protocol has been approved by the Research Ethical Committee of Longhua Hospital, affiliated to Shanghai University of Traditional Chinese Medicine, Shanghai, China (approval number: 2018LCSY058). All participants will be given sufficient time to reach a decision to sign the consent form prior to the study [9, 10]. And the protocol has been registered on Clinical Trials (ClinicalTrials.gov, ID: ChiCTR1900020519).

### Intervention

Because of the nature of the intervention, the patients and therapists could not be blinded to each other. The patients will be treated with Shi-style lumbar manipulations or mechanical lumbar traction, for 6 sessions over 2 weeks. The patients will be asked to note their drug use (including over-the-counter analgesics) after randomization.

### Shi-style lumbar manipulations

#### Tendon soothing

The therapist makes circular and rhythmic motions with the palm on the back of the waist for 3 min, and then presses with the palm root along the median line of the patient’s spine on both sides of the body for 3 min.

#### Osteopathic manipulation

The patient lies in the prone position. The assistant pulls the patient’s ankle back and upward, and then shakes the ankle up and down, while the therapist presses the waist of the patient lightly and flexibly for 3–5 times.

#### Collateral dredging

The therapist presses the bilateral sacrospinous muscles for 2–3 min, and then slightly shakes the patient’s ankles up and down with small amplitude and high frequency for 2 min.

### Mechanical lumbar traction

The traction treatment was based on the “Guidelines for diagnosis and treatment, and rehabilitation of DLS (2017)”. The patient receives 6 sessions of 30-min mechanical persistent lumbar traction over 2 weeks. The patient lies in a prone position. The traction angle was 0°. All procedures are performed with the patient’s comfort ensured. The traction force starts at 10 kg and increases by about 2 kg each visit, depending on centralization or reduction of symptoms. The maximum force used is 20 kg.

### Randomization and allocation

After the screening, patients will be randomized into two groups with an allocation ratio of 1:1. The randomization will be generated via SAS PROC PLAN software (SAS Inc., Cary, NC, USA) by an independent third-party clinical research organization (Institute of Basic Research in Clinical Medicine, China Academy of Chinese Medical Science) and concealed from the researchers by a senior data manager who is not involved in the study. The group assignment list will be sealed in opaque envelopes and be opened by the researchers following informed consent procedures and baseline testing.

### Blinding

All the investigators, physicians, nurses, assessors, analysts, and participants will be blinded to the group assignment until the end of the trial, when all statistical analyses are finished. The patients will not be blinded. If, after the first administration, any clinically significant adverse event potentially related to the treatment occurs, the study physician will re-evaluate the participant, and PI will decide whether the non-blinded procedure is necessary. If non-blinding is required, the allocation information will be provided.

### Outcome measurements

#### Primary outcome measurements

As pain is the most frequent complaint of DLS patients, we choose the VAS score as the primary outcome measurement to assess the low back pain and leg pain. VAS, a reliable and valid measurement of pain, has a horizontal, 100-mm-long line, with “no pain” recorded on the left side (score, 0) and “pain as bad as it could be” on the right side (score, 10). Patients are asked to place a hatch mark that corresponds to their current level of pain (both at rest and at most painful movement) on the line [11]. The VAS score is then determined by measuring the distance between the left endpoint and the point that the patient marked.

#### Secondary outcome measurements

The Oswestry Disability Index (ODI), one of the most widely used gold standards of outcome measurement in patients with low back pain, has been used for more than 25 years. ODI score is calculated by doubling the sum of the scores from the 10 sections and is reported as a percentage of the patient’s perceived disability.

Health-related quality of life will be measured using the SF-36 questionnaire, which is widely used to assess the quality of life for DLS patients. The SF-36 questionnaire is a tool for assessing physical status, consists of 36 items, and measures in seven dimensions: physical functioning, bodily pain, general health perceptions, physical role functioning, emotional role functioning, social role functioning, and mental health. It has been found reliable and valid in many patient populations [12, 13].

### Safety assessments

To assure the safety of participants, the data collection process will be supervised by the project director. At each visit, participants need to stay in the hospital for 30 min after the treatment and will be asked about the adverse effects for any signs of acute adverse reactions during the study period, such as lower limb paralysis or syndrome of cauda equina. Side effects will be recorded at each visit during treatment.

### Sample size calculation

We calculate the sample size according to our primary study. We conducted a preliminary clinical trial and pilot trial about Shi-style lumbar manipulations versus mechanical lumbar traction from January 2017 to May 2017. The primary efficacy parameter was the change of VAS scores from baseline to the end of 8-week treatment. Based on the previous results, we found that the primary efficacy parameter of Shi-style lumbar manipulations group increased by 4.56 and that of the mechanical lumbar traction group increased by 2.34. According to the formula of the rate in completely random design, *n*_1_ = *n*_2_ = $\frac{\left[{u}_{\alpha /2}\sqrt{2\overline{p}\left(1-\overline{p}\right)}+{u}_{\beta}\sqrt{p_1\left(1-{p}_1\right)+{p}_2\left(1-{p}_2\right)}\right]}{{\left({p}_1-{p}_2\right)}^2}$, among which, *n*_1_ and *n*_2_ were the number of patients in Shi-style manipulations group and the mechanical traction group respectively, *u*_*α*/2_ = 1.96 when type 1 error is 0.05, and *u*_*β*_ = 1.282 when type II error is 0.1 in two-sided tests. $\overline{p}$ is the mean of *p*_1_ and *p*_2_ [14]. A two-sided 5% significance level and 90% power in detecting treatment differences were considered, and the above relevant data were input into SPSS 20.0 software. This number of patients actually provided less than 80% power, considering an estimated dropout rate of 20%. Therefore, we will recruit a total of 72 patients with 36 patients in each group.

### Statistical analyses

Prior to all analyses, a detailed statistical analysis protocol will be developed. All data will be analyzed in the clinical research center of Longhua Hospital affiliated to Shanghai University of TCM by statisticians blinded to allocation using the SPSS 20.0 statistical software (SPSS Inc., Chicago, Illinois). Efficacy and safety analyses will be conducted according to the intention-to-treat principle using the “last observation carried forward” rule. Before randomization, baseline characteristics will be collected as descriptive statistics for each patient, including gender, age, BMI, duration of symptoms, and degree of lumbar spondylolisthesis. The data analysis of the primary outcome is based on the per-protocol population as a supportive analysis. Mean, standard deviation, median, quartiles and inter quartiles for continuous variables, and frequency for categorical variables will be calculated. Continuous variable followed the normal distribution will be presented as means with standard deviations (SDs) and calculated by an independent sample Student’s *t* test; otherwise, the data will be expressed as medians with ranges, and non-parametric tests will be used. Categorical variables will be expressed as number (%) and analyzed by *χ*^2^ test or Fisher’s exact test. Repeated measurement analysis of variance will be used to analyze value changes of VAS, ODI, and SF-36 scores in the different time points (baseline, week 1, 2, 4, 12, and 24) in the test. A *P* value of less than 0.05 is defined as statistical significant with two-sided 90% confidence intervals (CIs). Missing data will be input with the last observed response carried forward for all measures using the “last-value-carried-forward” principle.

### Data collection and monitoring

This is a 24-week clinical trial in which participants need to take research intervention for 2 weeks with 24 weeks’ follow-up. Five rounds of disease activity assessments (at baseline and at 2, 4, 12, and 24 weeks) and six rounds of safety assessments (at baseline and at 1, 2, 4, 12, and 24 weeks) will be recorded in Epidata (Version 3.1) by two statisticians independently. Disagreement will be solved by discussion and a third statistician. Longhua Hospital affiliated to Shanghai University of Traditional Chinese Medicine is responsible for monitoring and quality control.

### Quality control

Prior to the clinical trial, we will carry out unified training to make sure all the physicians, nurses, and assessors involved fully apprehend the process of the entire trial. To guarantee the quality of the whole trial, two supervisors will be sent twice a month to assure whether (1) all participants meet the inclusion criteria and do not meet the exclusion criteria, (2) study group recruits enough participants in the plan, (3) and all the participants fully follow the clinical trial process, and the case report form (CRF) has been completed. The standard operating procedures (SOP) will be invariably followed. Drop-outs, withdrawals (and the reasons), and any compliance of all patients occurring will be recorded in detail by the inspectors throughout the treatment and follow-up period.

## Discussion

DLS is a common spinal disease. Currently, there is not enough evidence based on the strict clinical trial of evidence-based medicine (EBM) because of the poor quality of current studies, for example, small sample size, no description of methods for randomization, and no standardized protocol which may lead to performance bias. Based on the theory of dynamic and static disequilibrium in TCM, manipulations have been proved more effective in treating chronic low back pain than other therapies such as analgesics, NSAIDs, or muscle relaxants. SLM, as one traditional Chinese exercise, has shown its efficacy in relieving DLS symptoms.

To our best knowledge, our study is the first elaborately designed, randomized, controlled trial to investigate the efficacy of manipulations in treating symptomatic DLS with clear standards. It is designed as a comprehensive study of pain relief, functional outcome, and adverse effects. Outcome measurements are widely used in research of symptomatic DLS to establish baselines, evaluate the effect of an intervention, and motivate patients’ self-evaluation. The VAS measurements, which have been found valid, reliable, and easy to apply in researches, are often used as the criterion standard to evaluate the pain intensity. The SF-36, a self-administered questionnaire, has been widely adopted as the criterion standard to estimate disease activity for its reliability and validity. Therefore, for the self-effectiveness assessment, we use the VAS for self-assessment of pain and SF-36 for self-assessment of function [15, 16]. The safety of SLM will be assessed by patients’ self-reporting of their quality of life and symptoms, such as lower limb paralysis or syndrome of cauda equina.

With the objective in mind, we have conducted a systematic literature search to guarantee the comprehensiveness of the trial before we begin this trial. We have thoroughly searched PubMed, MEDLINE, EMBASE, Cochrane Library, ISI web of knowledge, Wan Fang Data, CNKI databases, Vip Journal Integration Platform (VJIP), and Chinese BioMedical databases from the inception to December 2016. There has been no study pertaining to the efficacy and safety of the Shi-style manipulation in the treatment for patients with symptomatic DLS. The lack of good quality RCTs in the field leaves us with notable gaps in our knowledge, and in clinical practice, many decisions have to be taken without the benefit of high-quality evidence. So, we decide to conduct a prospective, randomized, controlled clinical trial, which also ensures the compliance of participants and meets ethical considerations, to closely detect the clinical efficacy of this chiropractic manipulation. The present study is built on our preliminary open experiment: a small-sample-sized, randomized, and controlled trial of SLM for symptomatic DLS with 1-month follow-up. The result of the preliminary trial has showed that SLM could better relieve the symptoms than celecoxib. We hope that this trial, with a larger number of patients, can provide adequate statistical power to further analyze and explore the efficacy of SLM. We decide to follow up the participants for 6 months after treatment. Thus, quality control is vital to the whole study, as we described in the protocol. To perform a reliable study, we will carry out unified training to make sure all the physicians, nurses, and assessors involved in the trials fully understand the process and details of SLM before the clinical trial. We also emphasize the choice of the main endpoint. Weekly frequency of SLM is the primary endpoint, which is a clinically meaningful measurement to reflect the relief of symptom. Moreover, for accurate assessment of the manipulation effect, we intend to focus on a specific population through this trial. First, we will exclude patients with low back pain of other causes rather than DLS, such as surgery, rheumatism, or severe osteoporosis, because manipulations may not be helpful in these conditions according to the previous investigation. Second, we will exclude patients with other serious conditions like heart failure and kidney failure to ensure SLM would not exert adverse effects on them.

This study is designed to investigate whether the SLM is preferred in treating symptomatic DLS, compared with mechanical lumbar traction. If this trial succeeds, it will provide an option of chiropractic manipulations for the patients and physicians as a better disease remission.

Hopefully, this trial will produce high-quality evidence pertaining to the efficacy and safety of the SLM in treating low back pain. The results will aid in clinical decision-making for the management of symptomatic DLS and provide useful information that can be incorporated into future guidelines.

## Additional file

Additional file 1: SPIRIT 2013 Checklist: Recommended items to address in a clinical trial protocol and related documents. (DOC 135 kb)

## Data Availability

The datasets used and/or analyzed during the current study are available from the corresponding author on reasonable request.

## Abbreviations

DLS: Degenerative lumbar spondylolisthesis
SLM: Shi-style lumbar manipulation
